# Ataxin-2: From RNA Control to Human Health and Disease

**DOI:** 10.3390/genes8060157

**Published:** 2017-06-05

**Authors:** Lauren A. Ostrowski, Amanda C. Hall, Karim Mekhail

**Affiliations:** 1 Department of Laboratory Medicine and Pathobiology, University of Toronto, Toronto, ON M5G 1M1, Canada; lauren.ostrowski@mail.utoronto.ca (L.A.O.); amandac.hall@mail.utoronto.ca (A.C.H.); 2 Canada Research Chairs Program, Faculty of Medicine, University of Toronto, 1 King’s College Circle, Toronto, ON M5S 1A8, Canada

**Keywords:** RNA metabolism, RNA-DNA hybrids, stress granules, ALS, SCA2

## Abstract

RNA-binding proteins play fundamental roles in the regulation of molecular processes critical to cellular and organismal homeostasis. Recent studies have identified the RNA-binding protein Ataxin-2 as a genetic determinant or risk factor for various diseases including spinocerebellar ataxia type II (SCA2) and amyotrophic lateral sclerosis (ALS), amongst others. Here, we first discuss the increasingly wide-ranging molecular functions of Ataxin-2, from the regulation of RNA stability and translation to the repression of deleterious accumulation of the RNA-DNA hybrid-harbouring R-loop structures. We also highlight the broader physiological roles of Ataxin-2 such as in the regulation of cellular metabolism and circadian rhythms. Finally, we discuss insight from clinically focused studies to shed light on the impact of molecular and physiological roles of Ataxin-2 in various human diseases. We anticipate that deciphering the fundamental functions of Ataxin-2 will uncover unique approaches to help cure or control debilitating and lethal human diseases.

## 1. Introduction

Two decades ago, a number of seminal studies identified the genetic cause of the debilitating neurodegenerative disease known as spinocerebellar ataxia type II (SCA2) [1,2,3]. SCA2 arises as a result of mutations within the *ATXN2* gene, which encodes the ATXN2 (also known as Ataxin-2) protein [1,2,3]. At the time, the molecular functions of Ataxin-2 were largely unknown. Within the first exon of *ATXN2* lies a trinucleotide repeat site consisting of tandemly-repeated CAG sequences, interrupted by CAA sequences, that form a polyglutamine (polyQ) tract [1,2,3]. In healthy individuals, the *ATXN2* polyQ tract generally consists of ~22–23 glutamines (Qs), with a sequence of (CAG)_8_CAA(CAG)_4_CAA(CAG)_8_. However, significant expansion of this tract to ≥34 Qs is the genetic cause of SCA2, although a few late-onset cases have been caused by slightly smaller expansions [1,2,3,4]. Intermediate expansion of the ATXN2 polyQ tract was later found to be associated with autosomal-dominant Parkinson’s disease [5]. A decade later, certain polyQ expansions of *ATXN2* were also linked to the devastating and too often fatal motor neuron disease known as amyotrophic lateral sclerosis (ALS) [6]. Specifically, intermediate expansion of the polyQ tract in the range of ~27–33 Qs was identified as a significant risk factor that is present in ~4.7% of ALS cases (patient cohort represents mix of familial and sporadic cases) [6].

**Figure 1:**
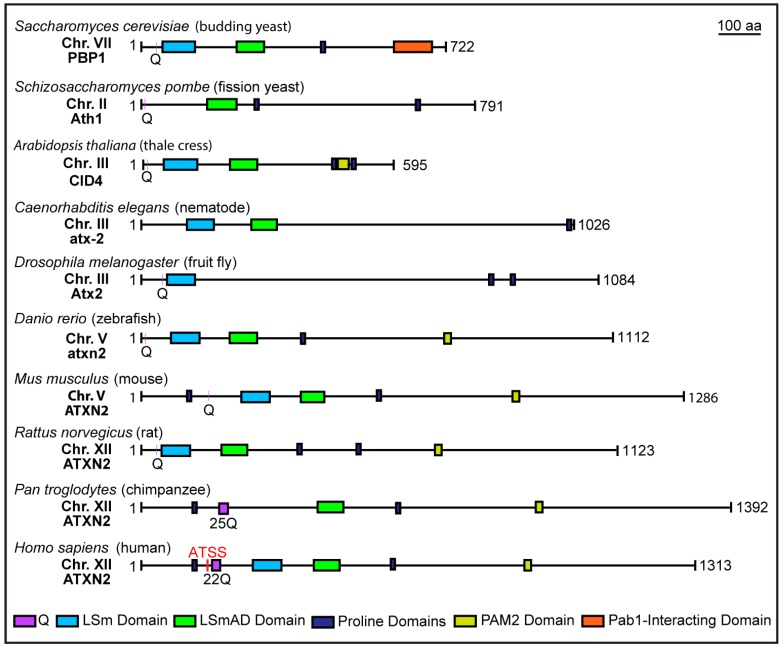
Structure of Ataxin-2 and its conserved domains across model species. The RNA-binding domains of Ataxin-2, Like-Sm (LSm) and LSm-associated (LSmAD), are located at the N-terminal region and are conserved across species. The C-terminal region of Ataxin-2 harbours a PAM2 domain, which interacts with the poly(A)-binding protein (PABP1). A polyglutamine (polyQ) tract is also located in the N-terminal of most mammalian species. For species with multiple Q residues upstream of the LSm/LSmAD domains (*S. pombe*, *D. rerio*, *M. musculus*), the Q represented was chosen based on amino acid sequence similarities with the regions flanking the polyQ site in human ATXN2. Conserved proline-rich domains are also depicted (consensus sequences: PPAXPTXXSP and PPSRPSRPPS). ATSS = alternative translational start site.

Human *ATXN2* is encoded within 25 exons and occupies ~147 Kb of genomic space on the minus strand of chromosome 12q24 [7]. Consisting of 1311 amino acid residues, the molecular weight of the mature peptide is ~140 kDa; however, additional studies employing reporter constructs have detected the use of an alternative start codon, from which translation produces a ~124 kDa peptide [7,8]. ATXN2 is a member of the Like-Sm (LSm) protein family, which participate in a large number of functions related to RNA processing and RNA metabolism [9]. Downstream of the polyQ site within the N-terminus, ATXN2 harbors an LSm domain and an LSm-associated domain (LSmAD; Figure 1). These are thought to be important, as the *ATXN2* gene is evolutionarily conserved from yeast to RNA-binding domains mediate physical interactions between LSm proteins and their target RNAs [9]. In addition to these domains, ATXN2 contains conserved proline-rich domains as well as a C-terminal PAM2 domain, which mediates interactions with the poly(A)-binding protein (PABP1) [10,11,12,13]. ATXN2 functions mammals (Figure 1).

ATXN2 is ubiquitously expressed. Although widely expressed in the mammalian nervous system, an already heightened expression in cerebellar Purkinje cells further increases with age [2,14]. Early studies suggested an exclusively cytoplasmic localization for ATXN2, but recent findings provide stronger support for a nuclear/cytoplasmic localization and suggest that ATXN2 may be shuttled to the nucleus to help regulate transcription [1,15,16]. At the subcellular level, ATXN2 localizes to the nucleolus, nucleoplasm, Golgi apparatus, rough ER membranes, and the receptor endocytosis machinery at the plasma membrane [17,18]. ATXN2 also associates with polyribosomes and is typically enriched in stress granules under environmental stress conditions [19]. These characteristics, in addition to the presence of RNA-binding domains, point to roles for ATXN2 in RNA processing and/or the regulation of translation.

In the past decade, numerous studies have set out to determine the function(s) of ATXN2 in order to shed light on its impact on human health and disease. Here, we present a detailed review of the current literature regarding the molecular functions of ATXN2 proteins in several model species, highlighting diverse roles ranging from the promotion of mRNA stability/translation, to the suppression of deleterious non-coding RNA (ncRNA)-harbouring R-loops, and to physiological roles in metabolism and circadian rhythmicity. Finally, we discuss how clinically focused studies are providing important insights into known functions of ATXN2 proteins while even pointing to new roles for the protein in the eukaryotic cell.

## 2. Wide-Ranging Molecular Functions of Ataxin-2

### 2.1. RNA Processing

Several roles have been uncovered for Ataxin-2 proteins within the broad functional category of RNA regulation. Studies with yeast, worms, flies, mice and humans have uncovered key molecular roles for Ataxin-2 proteins in the regulation of RNA stability/translation, suppression of deleterious R-loop accumulation, and cellular stress responses.

#### 2.1.1. mRNA Stability and Translation

Recent research has identified a role for Ataxin-2 proteins in regulating global mRNA stability and translation. Although ATXN2 harbours known RNA-binding domains and was thus suspected to be an RNA-binding protein (RBP) for some time, it was not until recently that direct binding of ATXN2 to over 4000 RNA molecules was demonstrated [20]. Photoactivatable-ribonucleoside-enhanced crosslinking and immunoprecipitation (PAR-CLIP) followed by high-throughput sequencing revealed direct binding of human ATXN2 to target RNAs in a poly(A)-binding protein 1 (PABP1)-independent manner [20]. ATXN2 binds to uridine-rich elements within the 3′ UTRs of its targets through its LSm domain, stabilizing target mRNAs and increasing their protein abundance [20]. Gene ontological (GO) analyses revealed that ATXN2 predominantly upregulates proteins involved in different aspects of RNA regulation including RNA splicing, polyadenylation and 3′ end processing [20]. Several of its targets include disease-associated RBPs, such as TAR DNA-binding protein 43 (TDP-43; ALS), CCR4-NOT transcription complex subunit 1 (CNOT1; iritis), Heterogeneous nuclear ribonucleoproteins A2/B1 (hnRNPA2B1; inclusion body myopathy), ELAV Like neuron-specific RNA-binding protein 2 (ELAVL2; Parkinson’s disease) and PABP1 (oculopharyngeal muscular dystrophy) [20]. Interestingly, either polyQ expansion or deletion of the polyQ domain prevents human ATXN2 from sustaining optimal protein production from a reporter gene [20]. This is consistent with a role for the polyQ domain of ATXN2 in promoting mRNA stability, although it does not rule out a potential role in the more direct promotion of protein synthesis. Future work should clarify the role of ATXN2 in the regulation of RNA stability and protein production in an endogenous system rather than a reporter-based model.

A potential mechanism through which ATXN2 proteins may promote mRNA stability comes from studies with the budding yeast *Saccharomyces cerevisiae*. These studies suggest that the yeast ATXN2 ortholog (Pbp1) promotes mRNA stability via modulation of RNA polyadenylation [21]. Pbp1 functions in the nucleus to regulate mRNA stability by promoting transcript polyadenylation [21]. The poly(A) tail alters mRNA stability by influencing translation initiation and/or the rate of mRNA decay [22]. Two proteins key to the regulation of yeast poly(A) tails include the PABP1 ortholog (Pab1) and the poly(A) nuclease (PAN) [23]. Pab1 binds to poly(A) tails and modulates their length by recruiting PAN to trim the tails prior to export [23]. In *PBP1* knockout cells, the 3′ termini of yeast pre-mRNAs still undergo proper cleavage but have shortened poly(A) tails [21]. Consistently, Pbp1 is a negative regulator of PAN [24]. It is also thought that Pbp1 may promote polyadenylation by Pab1 [21]. These findings indicate that Pbp1, the yeast ortholog of ATXN2, may promote mRNA stability and subsequently protein production by supporting mRNA polyadenylation.

Taken together, these studies in human and yeast cells suggest that Ataxin-2 proteins function to increase mRNA and protein levels, especially of factors involved in RNA control. This is accomplished by Ataxin-2 through direct binding to its targets and likely involves the regulation of polyadenylation. In addition, cases exist where Ataxin-2 proteins promote the production of certain proteins without altering their mRNA levels (e.g., see Ref. [25]), suggesting roles in translation.

In addition to yeast and human, research with other species also implicates Ataxin-2 proteins in the positive, as well as negative, regulation of translation. ATXN2 mouse ortholog knockout (Atxn2-KO) mice exhibit global reductions in protein synthesis, suggesting that Atxn2 promotes general translation [26]. However, Atxn2-KO mice exhibit increased mRNA and protein levels of a number of factors associated with ribosomal/translational control, including translation modulators Lsm12/Paip1 and apolipoprotein modulators Plin3/Mttp [26]. This suggests that in the absence of the translation-promoting Atxn2, key factors of the translational machinery may be upregulated in an effort to compensate for the reductions in global protein synthesis [26]. Despite this apparently broader role in the promotion of global protein synthesis, Atxn2 can also repress the translation of at least some mRNAs. For example, mouse studies suggest a role for Atxn2 in the repression of mRNA translation with implications in synapse-specific plasticity associated with long-term memory [27]. More specifically, Atxn2 functions as part of the machinery required for optimal translational repression by several microRNAs, which include known miRNA-pathway proteins Argonaute (AGO1) and the Me31B RNA helicase [27]. A similar role has been identified for the *Drosophila* ATXN2 ortholog (dATX2), which functions with the RBP dFMR1 (fragile X mental retardation protein 1) in translational control in the nervous system to mediate long-term olfactory habituation [28]. Yet dATX2 may also promote translation. For example, mutations disrupting the expression of dATX2 mimic the loss of actin synthesis-promoting factors leading to defective actin filament formation as well as related tissue loss/degeneration, female sterility, and lethality [29]. But unlike actin regulators, dATX2 does not directly associate with actin filaments. Thus, the role of dATX2 in the promotion of actin filament formation is likely indirect and may be at the level of promoting the mRNA stability and/or translation of actin regulatory factors. Consistent with this rationale, other research employing *Drosophila* indicates that dATX2 physically assembles with polyribosomes, as does human ATXN2 [30]. *Drosophila* and human Ataxin-2 are not components of the ribosomes themselves, but assemble into messenger ribonucleoprotein (mRNP) complexes [30]. This is mediated by the LSm, LSmAD and PAM2 domains through distinct mechanisms. The LSm/LSmAD domains are sufficient but not necessary for direct dATX2 assembly with polyribosomes, and the PAM2 domain is required for dATX2 binding to polysome-bound dPABP1 [30]. Interestingly, SCA2-like polyQ expansion (58Q) does not alter polyribosome assembly of dATX2, suggesting that the polyQ domain is likely not involved in polyribosome assembly [30].

Lastly, studies with the hermaphrodite *Caenorhabditis elegans* revealed a role for ATX-2, the worm ATXN2 ortholog, in the negative and positive regulation of translation [31]. Hermaphrodite germline development requires ATX-2, whose loss decreases stem cell proliferation and increases masculinization [31]. It was shown that these defects result from translational dysregulation. ATX-2 loss elevates the protein but not mRNA levels of GLD-1 (KH-domain female germline-specific tumor suppressor). Increased GLD1 levels leads to hyper-repression of genes such as glucagon-like peptide 1 (GLP-1), which is a component of the stem cell-sustaining Notch signaling pathway and is key to germline development [31]. Elevated GLD-1 also hyper-represses the spermatogenesis to oogenesis switch inducer TRA-2 (transformer-2 sex-determining protein), causing germline masculinisation [31]. On another front, ATX-2 loss decreases the protein but not mRNA levels of a protein called MEX-3 (muscle excess protein 3), which is typically expressed in the germline and represses the yolk receptor RME-2 [31]. Thus, ATX-2 loss decreases MEX-3 levels thereby aberrantly de-repressing RME-2 in the germline [31]. These studies in mice, flies and worms suggest that Ataxin-2 proteins have a conserved role in the regulation of translation.

Taken together, these findings highlight a role for Ataxin-2 proteins in the regulation of mRNA stability and translation in several species. Ataxin-2 promotes mRNA stability in both PABP1-dependent and independent manners, and functions to promote protein synthesis at the post-transcriptional and translational levels. Although most studies suggest Ataxin-2 proteins function to promote global translation, some have shown that Ataxin-2 proteins also function to repress translation of select mRNAs [26,28]. Therefore, Ataxin-2 proteins appear to play a complex role in translational regulation.

#### 2.1.2. Stress Granules and Processing Bodies

RNA processing is critical to gene expression, genome stability, and cellular responses to stressful environmental stimuli including heat and osmotic shock. Under stress conditions, various cellular processes are altered in order for cells to adapt and maintain cellular integrity. Translation is often aborted during stress, giving rise to two distinct cytoplasmic sites of mRNA triage known as stress granules and processing-bodies (P-bodies) [32,33]. At the heart of these stress response pathways are various RBPs including human PABP1 and its yeast ortholog Pab1 [34,35]. In addition, from yeast to human, Ataxin-2 proteins play a role in response to different environmental stresses [19,34,36,37]. Heat shock promotes stress granule formation in mammalian cells [34]. Heat shock promotes stress granule formation in mammalian cells [34]. Under these stress conditions, immunofluorescence revealed cytoplasmic co-localization of PABP1 and ATXN2 in stress granules [34]. Furthermore, siRNA-mediated knockdown of human ATXN2 decreases the number and size of stress granules while yeast *PBP1* knockout decreases stress granule formation under glucose deprivation [19,36]. These findings suggest that ATXN2 and its orthologs mediate cellular responses to stress by promoting stress granule formation.

Studies have shown that P-bodies can co-localize with stress granules under certain stress conditions [38,39]. Since ATXN2 proteins are stress granule components and P-bodies can localize to stress granules, it is possible that Ataxin-2 may also have a direct or indirect connection with P-bodies. Yeast two-hybrid experiments revealed that human ATXN2 physically interacts with the key P-body component RNA helicase DDX6, and confocal immunofluorescence microscopy indicated that ATXN2 and DDX6 co-localize under stress [19]. In yeast, Pbp1 physically interacts with the DDX6 ortholog Dhh1, suggesting that ATXN2-P-body connections may be evolutionarily conserved [40]. Further analysis showed that overexpression of human ATXN2 disrupts DDX-6-marked P-bodies causing them to appear less defined and more diffuse [19]. As DDX6 is an essential component of P-bodies, the role of ATXN2 in P-bodies appears to be through an unknown mechanism that recruits DDX6 [41]. Importantly, decreasing ATXN2 levels does not alter P-bodies suggesting that ATXN2 is not required for P-body assembly, while increasing ATXN2 levels disrupts DDX6 recruitment and ultimately P-body formation [19]. Moreover, ATXN2 levels inversely correlate with PABP1, another component of stress granules, as the overexpression and knockdown of ATXN2 leads to a decrease and increase of PABP1 levels respectively, thereby suggesting that the former is a dosage-dependent regulator of the latter [19]. As ATXN2 levels are altered in various human diseases, consequent disruption of stress granules and P-bodies may contribute to pathobiological processes underlying such diseases. The reported impact of ATXN2 proteins on general RNA processing and cellular stress responses suggests that ATXN2 proteins can indirectly regulate a high number of cellular processes.

#### 2.1.3. R-Loop Regulation

The RNA-binding capabilities of Ataxin-2 proteins are not limited to protein-coding mRNAs. Ataxin-2 has been shown to preserve genome integrity by binding to ncRNAs and preventing them from promoting the deleterious accumulation of nucleic acid structures known as R-loops [15,42]. During transcription, nascent transcripts can reinvade the DNA duplex and anneal to template DNA, giving rise to a three-strand nucleic acid structure known as an R-loop, consisting of an RNA-DNA hybrid and a displaced single-stranded DNA (reviewed by [43]). Yeast chromatin immunoprecipitation (ChIP) employing an antibody that recognizes RNA-DNA hybrids in a sequence-independent manner revealed that loss of the yeast Ataxin-2 protein (Pbp1) leads to an increase in R-loop accumulation in the intergenic spacer (IGS) regions separating ribosomal DNA (rDNA) repeats [42,44]. Pbp1 lacking the Pab1-binding domain retained full R-loop-suppressing capacity [42]. R-loop accumulation stalls DNA replication forks leading to aberrant recombination events within rDNA repeats and eventually shortening replicative lifespan [42]. RNA immunoprecipitation revealed that Pbp1 binds IGS ncRNAs and prevents their engagement in R-loops [42]. It is possible that ATXN2 proteins have an evolutionarily conserved role in the regulation of R-loop levels across different species. Indeed, human ATXN2 deficiency leads to genome-destabilizing R-loops accumulation [15]. Immunofluorescence experiments revealed that human ATXN2 indeed localizes to the cytoplasm, nucleus, as well as the rDNA-harbouring nucleolus, in which the localization of the ATXN2 protein is inversely correlated with R-loop levels [15].

Importantly, processes linked to caloric restriction can efficiently counter genome-destabilizing R-loop accumulations in yeast and human cells [15,42]. Specifically, yeast work revealed that caloric restriction activates the R-loop suppressing proteins Rnh1/201 (yeast RNaseH1/2) and Pif1 (ATP-dependent RNA helicase) [15,45]. RNaseH enzymes can degrade the RNA component of RNA-DNA hybrids within R-loops [46,47]. Pif1 enzymes can resolve RNA-DNA hybrids and unwind R-loop-stabilizing structures known as G-quadruplex DNA (G4DNA), which can be present on the displaced single stranded DNA component of R-loops [48,49]. Specifically, this action of caloric restriction is accomplished by its ability to increase by ~20% the levels of intracellular magnesium (Mg^2+^), a cofactor for both RNaseH1 and Pif1 [15]. In Pbp1-deficient yeast cells and ATXN2-deficient human cells, direct Mg^2+^ supplementation counters R-loop accumulation and its related genome instability [15]. Thus, if R-loop accumulation emerges as a driver of pathogenicity in ATXN2-linked neurodegenerative diseases, magnesium supplementation and small molecule modulators of magnesium transporters and/or R-loop levels should be tested as potential therapeutic strategies within these clinical settings [50,51].

### 2.2. Physiological Functions

The existence of diverse, yet evolutionarily conserved, molecular functions for Ataxin-2 proteins is consistent with the fact that they do impact numerous physiological processes. For example, Ataxin-2 is involved in embryonic development, apoptosis, actin development, cellular proliferation, insulin signaling in obesity, and various metabolic processes to name a few (Table 1). Such impacts for Ataxin-2 proteins may be driven by its target mRNAs, R-loop regulation at various genetic loci, or modulation of stress granules and P-bodies. In this section, we highlight the functions of Ataxin-2 related to TORC1 signaling and circadian rhythms. For a review focusing on the function of Ataxin-2 in cell metabolism, we refer the reader elsewhere [52].

#### 2.2.1. Target of Rapamycin

The target of rapamycin (TOR or mTOR in mammals) functions in two distinct complexes: TOR complex 1 (TORC1) and TOR complex 2 (TORC2) (reviewed in [69]). This protein kinase regulates cellular growth processes in response to nutrient availability and is sensitive to the immune-suppressant drug rapamycin. Yeast cells expressing overactive TORC1 are resistant to the TORC1 inhibitors rapamycin and caffeine; however, Pbp1 overexpression restores TORC1 sensitivity to both inhibitors [53]. Importantly, Pbp1 overexpression does not alter TORC1 assembly, suggesting that Pbp1 may inhibit TORC1 activation and not its expression [53]. Indeed, TORC1 activity, as revealed by monitoring phosphorylation of the TORC1 target Sch9, is reduced in cells overexpressing Pbp1 [53,70]. Fluorescence microscopy revealed that both Pbp1 overexpression and heat stress promote the sequestration of TORC1 within stress granules [53]. Thus, under either heat stress or in environmentally non-stressed conditions but with ectopically increased levels of the yeast Ataxin-2 protein Pbp1, TORC1 is inhibited via its sequestration within stress granules.

TORC1, AMP-regulated kinase (SNF1) and PAS kinase (PSK1) are three evolutionarily conserved nutrient sensing kinases that exhibit functional crosstalk and coordinate cellular energy and metabolism [71,72]. This crosstalk raises the possibility that Pbp1/stress granule-dependent inhibition of TORC1 may involve yeast Snf1 and/or Psk1. Moreover, both yeast two-hybrid and co-purification analyses identified Pbp1 as a binding partner of Psk1 [73]. However, Psk1 is only able to phosphorylate truncated Pbp1 lacking the N-terminal 196 amino acids [54]. Specifically, Psk1 associates with the 420–722 amino acid region of Pbp1 whilst the 1-97 N-terminal region inhibits this interaction. Psk1 phosphorylates a threonine residue within Pbp1 leading to Pbp1 activation, stress granule formation, and consequently the inhibition of TORC1 [54]. Psk1 itself is phosphorylated/activated by Snf1. Thus, there exists a cross talk, in which Snf1 phosphorylates/activates Psk1, which in turn phosphorylates/activates Pbp1 allowing it to sequester/inhibit TORC1 within stress granules.

Roles for other Pbp1/ATXN2 orthologs in nutrient sensing and metabolic pathways have also been reported. In mice, obesity markers branched-chain amino acids (BCAAs) are strong stimuli of mTORC1 (Reviewed in [74]). Levels of proteins that metabolize these mTORC1-activating BCAAs are decreased upon loss of mouse Atxn2 [75]. Additionally, Atxn2 levels increase under nutritional stress and knockdown of mouse Atxn2 in this same setting leads to an increase in the phosphorylation of mTOR targets suggesting that Atxn2 inhibits mTOR signalling [56]. In *Drosophila*, dATX-2 regulates organismal size by operating upstream of and limiting mTOR signalling [55]. Thus, roles for Ataxin-2 proteins in the modulation of cellular metabolism and stress responses rely on controlling stress granules and TOR signaling.

#### 2.2.2. Circadian Rhythmicity

Ataxin-2 proteins can play key roles in the function of the circadian clock, an endogenous day-night cycle in which different processes oscillate and are synchronized, from physiology to behavior [76]. In *Drosophila*, a critical component of the circadian pacemaker is the PERIOD protein dPER, which is regulated at multiple levels, from transcription to post-translational modification [77]. Two studies in *Drosophila* revealed that dATX2 is required for translation of per mRNA [57,58]. Knockdown of dATX2 lowers dPER levels and gives rise to a longer than normal period of constant darkness [57,58]. This phenotype can be rescued via overexpression of dPER. Mass spectrometry revealed that the dTYF (twenty-four) protein, which is required for translation of PER, interacts with both dATX2 and dPABP1 [58,78]. dTYF and dPABP1 bind dATX2 at different regions, and although loss of dPABP1 binding does not affect dTYF binding, it does result in alterations to dPER oscillations [57,58]. Interestingly, knockdown of dATX2 does not affect dTYF association with per mRNA. These findings suggest that dATX2 functions as an activator of translation by coordinating a dTYF/dPABP1 and per mRNP complex, the components of which are all required for dPER translation.

Although clock genes are conserved from fruit flies to mammals, mammalian clocks are much more complex, with three PERIOD proteins (PER1-3) [79,80]. Thus, the regulatory role of Ataxin-2 proteins in the circadian clock might not be conserved or might be altered between species. Indeed, a recent study in mice revealed apparently contrasting results for the role of Atxn2 in circadian rhythmicity [81]. In Atxn2-KO mice, PER1 and PER2 protein levels were not affected relative to wild-type mice indicating that Atxn2 is not an activator of translation of the PER1 and PER2 proteins [81]. However, after transient deregulation of the circadian cycle, Atxn2-KO mice took significantly longer to re-adjust their cycle compared to wild-type mice, suggesting that the clockwork is in fact impaired in the absence of Atxn2 [81]. Although the role of Ataxin-2 in circadian rhythmicity is more subtle in mammals than in *Drosophila*, an impact is still evident. Moreover, as ATXN2-linked diseases such as SCA2 show disturbances in rapid eye movement (REM) sleep, which is regulated by the circadian clock, the role of ATXN2 in circadian rhythmicity warrants further investigation [82].

**Figure 2:**
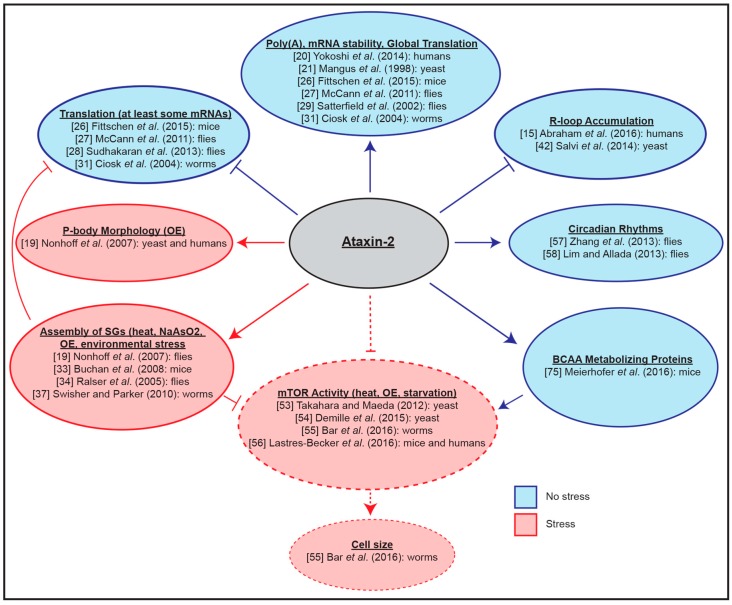
Functions of Ataxin-2 proteins under stressed and non-stressed conditions. Studies from a variety of model organisms demonstrate a role for Ataxin-2 proteins in the regulation of mRNA polyadentylation, stability, translation, R-loops, circadian rhythms, branched-chain amino acid (BCAA) metabolism, mTOR activity, stress granule (SG) assembly, and P-body morphology. Dashed lines represent indirect regulation by Ataxin-2. Reference numbers for papers are shown.

The biological functions of Ataxin-2 proteins discussed so far (summarized in Figure 2) likely only constitute a fraction of the full functional impact of these evolutionarily conserved proteins. Therefore, it is not surprising that ATXN2 dysfunction is linked to a vast array of human diseases. In the next section, we will highlight some insights from studies focusing on human diseases in which ATXN2 has been shown to contribute to pathogenicity.

## 3. Insights into Atxn2 Roles from Human Disease-Focused Studies

The functional studies outlined above have provided a wealth of information regarding the function of wild-type ATXN2 and its orthologs. However, key functional insights have also emerged from disease-focused studies employing human cells, non-human primate cells and mouse models. These studies identify a critical role for ATXN2 in cell death and calcium homeostasis. ATXN2 also cross talks with several disease-associated proteins, pointing to putative functions in autophagy, apoptosis, mRNP formation and nutrient signalling.

### 3.1. polyQ Expansion in Cell Death and Calcium Homeostasis

#### 3.1.1. Cell Death

Early disease-focused studies identified a role for ATXN2 in mediating cell death [17]. SCA2-like polyQ expansion of ATXN2 (58Q and 104Q) in primate and rat cell lines led to dispersion of the Golgi apparatus, apoptosis and caspase-3 activation [17]. Later studies confirmed this finding with monkey COS-1 and human HEK293T cells, showing that SCA2-like polyQ expansion (58Q and 108Q) led to cell death and Golgi dispersion [83]. Interestingly, N-terminal ATXN2 fragments truncated within the LSmAD domain but retaining the expanded polyQ tract are less cytotoxic than full-length polyQ-expanded ATXN2 [83]. This suggests that domains downstream of the polyQ tract are required for ATXN2-induced cell death. It is unknown whether the role of mutant ATXN2 in inducing cell death corresponds to a gain of toxic function or a loss of a normal function of the protein. However, two lines of evidence have emerged to tentatively suggest that polyQ expansion of ATXN2 may induce cell death through at least a loss of some wild-type function. First, polyQ expansion of disease-related proteins often results in the formation of intranuclear aggregates which are suspected to gain a toxic function, eventually leading to cell death [84,85]. However, some studies have shown this is not necessarily the case for cell death mediated by ATXN2 [17]. Specifically, the formation of aggregates is not required for ATXN2-mediated cell death as it is observed in both of the aggregate-forming ATXN2 104Q cells and aggregate-free ATXN2 58Q cells [17]. This supports previous findings using mouse models showing that intranuclear aggregate formation is not required for SCA2 pathogenesis [86]. Therefore, it is possible that ATXN2 polyQ expansion does not create a gain of toxic function, or that any gain of toxic function relates to something other than intranuclear protein aggregation. Interestingly, although intranuclear ATXN2 aggregates are not detected in SCA2 mice, cytoplasmic ATXN2 aggregation appears to drive SCA2 pathology [86]. This is uncharacteristic of disease-like polyQ proteins. Therefore, more work is required to establish the connection between cytoplasmic aggregates and polyQ-associated neurodegeneration. Additionally, polyQ expansion of Ataxin-2 increases its half-life, which may contribute to its gain-of-function toxicity [6]. Thus, disease-associated polyQ expansion can promote but is not required for ATXN2-dependent cell death. More work is needed to clarify if ATXN2 polyQ expansion induces cell death as a result of a loss and/or gain of function.

#### 3.1.2. Calcium Homeostasis

Since SCA2 primarily as well as factors in calcium homeostasis pathways [87]. Of note, both Atxn2-KO and Atxn2-CAG42-KI result in down-regulation of Itpr1, a calcium transporter, with Atxn2-CAG42-KI mice being affects Purkinje neurons, which are known integrators of calcium currents, it is consistent that a role for ATXN2 in regulating calcium homeostasis is recognized. Global transcriptome analysis comparing the profiles of Atxn2-KO mice and mice with knock-in of a 42Q version of Atxn2 (Atxn2-CAG42-KI) was conducted by microarray and validated by RT-qPCR [87]. Both Atxn2-KO and Atxn2-CAG41-KI mice displayed down-regulations of transcripts involved in lipid and growth signaling pathways, affected earlier [87]. Atxn2-KO and Atxn2-CAG42-KI mouse cerebella exhibited depletion of ITPR1 protein from the soluble fractions, while accumulation of membrane-associated ITPR was only observed in Atxn2-CAG42-KI mice [87]. Additional studies with mouse models found that pathologically expanded Atxn2 (58Q) physically interacts with InsP_3_R1, an intracellular calcium release channel; an interaction that is undetectable in Atxn2 wild-type cells [88]. In addition, the authors found that Atxn2-58Q transgenic mice demonstrate higher Ca^2+^ responses than wild-type mice, and that treatment with a Ca^2+^ stabilizer alleviated motor deficits. These findings point to a role for Atxn2 in the regulation of calcium flux and suggest that polyQ expansion of ATXN2 may impair calcium homeostasis through loss and gain of function.

Taken together, these disease-focused studies reveal a role for Ataxin-2 proteins in the regulation of processes such as cell death and calcium homeostasis. Future research should aim to elucidate the mechanism through which Ataxin-2 proteins contribute to these pathways in different cell-types and species. Future studies should also further investigate whether ATXN2 polyQ expansion results in loss and/or gain of function(s). Interestingly, it was recently reported that the therapeutic reduction of Ataxin-2 expression by antisense oligonucleotide therapy improves motor function and survival of SCA2 mice, further strengthening the argument that ATXN2 mutations may produce a gain of function [89,90].

### 3.2. ATXN2 Intersects with Various ALS-Related Factors

It is generally thought that ALS is not driven by a single genetic factor, but rather by a complex interplay between several disease-associated gene products [91]. To date, over 25 genetic loci have been linked to ALS [92]. ATXN2 has been shown to interact with a handful of these gene products, including C9ORF72, TDP-43 and FUS [91]. Several studies examining the link between ATXN2 and these ALS proteins have provided additional insight into the function of wild-type and mutant ATXN2, and how their synergistic effects can contribute to disease.

#### 3.2.1. C9ORF72

Although the function of the C9ORF72 gene product is unclear, *C9ORF72* is so far the most commonly mutated gene in familial ALS and frontotemporal dementia (FTD) [93,94]. *C9ORF72* mutations consist of intronic (GGGGCC)_n_ hexanucleotide repeat expansions (HRE) that have been proposed to contribute to ALS through at least three mechanisms [93,94]. These include (1) reduced C9ORF72 expression (loss of function), (2) accumulation of bidirectionally-transcribed (sense and antisense) toxic repeat ncRNAs and their sequestration of RBPs and/or (3) repeat-associated non-ATG (RAN) translation of toxic dipeptides [95]. An interesting link between C9ORF72 and ATXN2 has recently been proposed. In a French cohort consisting of ALS, FTD or FTD-ALS patients, a significant co-occurrence (23% of patients) was found for *C9ORF72* HRE and *ATXN2* intermediate-length CAG (29-33Q) expansions [96]. Strikingly, all of these individuals fell into the FTD-ALS group, tentatively suggesting that *C9ORF72* and *ATXN2* mutations may especially contribute to the development of FTD-ALS. On the molecular level, a synergistic effect of *C9ORF72* and *ATXN2* mutations on autophagy has been observed [97]. Loss of C9ORF72 function alone impairs autophagy and has a small but significant deleterious effect on neuronal survival [97]. However, C9ORF72 deficiency in cells also expressing ALS-linked expanded ATXN2 induces ATXN2 aggregation and exhibits a synergistic effect in terms of motor neuron dysfunction and neuronal cell death [97]. This points to a genetic interaction between *C9ORF72* and *ATXN2* in the regulation of autophagy and its impact on neuronal survival.

#### 3.2.2. TDP-43

TAR DNA-binding protein 43 (TDP-43) is a nuclear-cytoplasmic RBP that functions as a global regulator of gene expression at the transcriptional and post-transcriptional levels [98]. Thirty-eight dominant TDP-43 mutations have been identified in ALS patients [98]. Molecularly, these mutations may contribute to the development of the disease through several mechanisms including an abnormally predominant cytoplasmic localization and the formation of TDP-43 inclusions in the brain [99].

TDP-43 and ATXN2 can form a protein complex [6]. ATXN2-TDP43 interactions are likely RNA-dependent as they are abolished upon mutation of RNA-binding sites in TDP-43 [6]. In addition, ATXN2 has been identified as a strong modifier of TDP-43 toxicity in both cellular and organismal models [6]. Specifically, upregulation of Ataxin-2 proteins in yeast and flies increases the toxicity of wild-type and mutant TDP-43 and reduces fly lifespan, while loss of yeast Ataxin-2 suppresses TDP-43 toxicity [6]. From a disease-relevant standpoint, ATXN2 polyQ expansion increases ATXN2 stability and enhances its association with TDP-43 [6]. During heat stress, ATXN2 polyQ expansion increases the number of cells with mislocalized/cytoplasmic TDP-43 as well as discrete cytoplasmic ATXN2 foci [6]. Given the physical association of ATXN2 and TDP-43 even in wild-type cells, the proteins may function in a common pathway. In fact, recent research has identified the involvement of ATXN2 in caspase-3 activation and pathological TDP-43 modifications [100]. In HEK293T cells, ALS patient-derived lymphoblast cell lines and differentiated neuroblastoma cells (M17 cells), intermediate polyQ expansion of ATXN2 (31-32Q) induced caspase-3 activation under stress [100]. This increased C-terminal cleavage and phosphorylation of TDP-43, as well as accumulation of insoluble phosphorylated TDP-43 [100]. Interestingly, SCA2-like polyQ expansion (39Q) inhibits caspase-3 activation, and does not alter insoluble phosphorylated TDP-43 levels [100]. The authors suggest that this could imply ALS-linked intermediate polyQ expansion of ATXN2 enhances a normal function of ATXN2 (e.g., regulation of caspase-3 activation), whereas longer polyQ expansions associated with SCA2 disrupts this function. They present a model in which intermediate polyQ expansion of ATXN2 lowers the threshold for caspase activation under stress, leading to aberrant TDP-43 cleavage and initiating a pathological cascade that culminates in motor neuron death [100]. Additional connections between Ataxin-2 and TDP-43 were recently uncovered. Specifically, it was reported that disruption of Ataxin-2 expression reduces pathology in a TDP-43 mouse model of ALS [89,90].

#### 3.2.3. FUS

Several missense mutations in fused in sarcoma (FUS) were identified to contribute to ALS development [101,102]. In ALS patients, FUS mutation disrupts its normal nuclear localization in the nervous system resulting in cytoplasmic accumulations that trigger ER stress [101,102]. It remains unclear if FUS mutations result in a loss of function in the nucleus and/or a gain of toxic function in the cytoplasm.

ATXN2 was identified as a modifier of FUS pathology [103]. ATXN2 and FUS co-localize and co-precipitate in ALS patient motor neurons [103]. In addition, similar to TDP-43, FUS association with ATXN2 is strengthened by intermediate length polyQ expansion of ATXN2 [103]. Moreover, intermediate polyQ expansion of ATXN2 increases cytoplasmic FUS levels, ER stress, Golgi fragmentation, and apoptosis [103]. This provides further evidence that ATXN2 functions in cell death pathways. Additional studies identified a role for ATXN2 and FUS in mRNP granule formation [104]. Expression of mutant FUS recruits wild-type ATXN2 to mRNP granules, and overexpression of ATXN2 (regardless of polyQ length) inhibits FUS mRNP granule assembly [104]. Truncated TDP-43 was also found to recruit wild-type ATXN2 to mRNP granules, and overexpression of ATXN2 similarly inhibited TDP-43 mRNP granule formation [104]. These findings strengthen the argument that ATXN2 has a key role in FUS/TDP-43-containing mRNP granule formation, and that altering ATXN2 levels leads to an abnormal distribution of RBPs.

These studies identify intersecting roles for different ALS-associated proteins, implicating ATXN2 in complex and potentially pathological cascades. These findings also reveal that ATXN2 impacts several disease-related processes including autophagy, apoptosis and mRNP formation. We anticipate that future studies will solidify these links and identify additional roles for ATXN2 in processes that are typically perturbed in ALS/SCA2.

### 3.3. ATXN2 Dysfunction Is Linked to Several Human Diseases

In addition to causing SCA2 and increasing the risk of developing ALS, mutations in ATXN2 may play a role in a handful of other diseases, including Parkinson’s disease (PD), spinocerebellar ataxia type I (SCA1), Machado-Joseph Disease (SCA3), tauopathies, primary open-angle glaucoma (POAG), obesity and type I diabetes.

#### 3.3.1. Parkinson’s Disease

Global transcriptome profiling using cells derived from SCA2 and PD patients uncovered similar transcriptomic changes as compared to healthy controls [105]. The greatest alterations were related to factors known to bind RNA and poly(A) RNA [105]. SCA2 and PD similarities point to a potential overlap between pathological mechanisms and/or hallmarks. Accordingly, ATXN2 has been linked to the regulation of PD-relevant genes such as PTEN-induced putative kinase 1 (PINK1) [106,107,108]. PINK1 governs mitochondrial quality control, regulates selective autophagy and is mutated in PD [106,107,108]. Global transcriptome profiling of Atxn2-KO mouse cerebella identified Atxn2 as a strong modifier of PINK1 levels, such that Atxn2-KO severely downregulates PINK1 expression [109]. In contrast, in SH-SY5Y neuroblastoma cells under stress, knockdown of either ATXN2 or PINK1 enhanced the expression of the other [109]. This suggests that ATXN2 may positively regulate PINK1 levels during stress. The authors note that both ATXN2 and PINK1 appear to be simultaneously regulated, rather than ATXN2 acting upstream of PINK1. This suggests that their levels are regulated by the same upstream factor, and that they may play parallel roles in mitochondrial quality control. Further evidence of a role for Ataxin-2 in mitochondrial maintenance stems from recent global proteomic studies that identified broad mitochondrial dysfunction in Atxn2-KO mice [75].

#### 3.3.2. Spinocerebellar Ataxia Type I

Recent research has also linked mutations in Ataxin-2 to SCA1 [110]. It was demonstrated that Ataxin-1 physically interacts with fly dAtx2 and human ATXN2, a finding which has been verified by interaction network analyses [111]. Using a *Drosophila* model, it was shown that dAtx2 is a potent genetic modifier of SCA1 toxicity, such that neurodegeneration is enhanced by increased dAtx2 levels and suppressed by decreased dAtx2 levels, suggesting a gain of function [110]. Indeed, the study reported that pathological expansions of Ataxin-1 induce nuclear accumulation of Ataxin-2, which causes severe eye degeneration.

#### 3.3.3. Machado-Joseph Disease

polyQ expansion of ATXN2 has also recently been linked to the age-at-onset of a neurodegenerative disease closely related to ALS/SCA2, known as spinocerebellar ataxia type III (SCA3) or Machado-Joseph’s disease (MJD) [112,113,114]. Three studies have linked the length of the ATXN2 polyQ tract to age-at-onset of MJD, such that intermediate-length alleles correspond to an earlier age-at-onset in both European and Chinese cohorts [112,113,114]. This points to potentially overlapping pathogenic mechanisms in ALS and MJD. Although it has been suggested that Ataxin-2 is a genetic modifier of numerous neurodegenerative diseases, further research is needed to clarify the role of Ataxin-2 in PD and MJD, as well as other neurodegenerative diseases [115].

#### 3.3.4. Tauopathies

One study to date has linked Ataxin-2 to tauopathies [116]. By employing a *Drosophila* model, the authors showed that dAtx2 is an enhancer of Tau toxicity [116]. However, no follow up studies have clarified this association, and thus future work is required to determine if Ataxin-2 proteins in fact play a role in the development of tauopathies.

#### 3.3.5. Primary Open-Angle Glaucoma

Considering Glaucoma a neurodegenerative disease is a recent hypothesis that is postulated will help elucidate mechanisms behind neural injury in the visual system [117,118]. Interestingly, mutation of ATXN2 has been associated with an increased susceptibility to POAG [119]. However, rather than the more widely researched polyQ mutation of ATXN2, it has been discovered that four single nucleotide polymorphisms (SNPs) are associated with POAG [119]. It remains unclear if these SNPs trigger a loss and/or gain of ATXN2 function.

#### 3.3.6. Obesity and Type I Diabetes

Consistent with the apparent role of ATXN2 in regulating nutrient signaling and metabolism, ATXN2 polyQ expansion has been linked to obesity and type I diabetes [120]. In a few European individuals with SCA2, pathological manifestations in the middle stages of the disease included polyphagia (excessive hunger), obesity, and type I diabetes [120]. Studies using a mouse model fed a fat-enriched diet showed that Atxn2-KO mice experience an increase in weight gain compared to normal controls [121]. The link between ATXN2, obesity and diabetes was corroborated by a later study in which Atxn2-KO mice also exhibited obesity and hepatosteatosis [63]. This could be attributed to altered insulin signaling, as these mice also exhibited reduced insulin receptor expression and increased insulin levels [63]. Large-scale proteomic/metabolomic studies of Atxn2-KO mice livers and cerebella reveal gross alterations in pathways related to nutrition and basal metabolism, including the modulation of branched chain or other amino acid metabolism, fatty acids and citric acid cycle [75]. This suggests a role for ATXN2 in maintaining energy balance. Although the link between ATXN2 and obesity/diabetes is intriguing, research employing a larger cohort is needed in order to clarify the significance of these findings.

**Figure 3:**
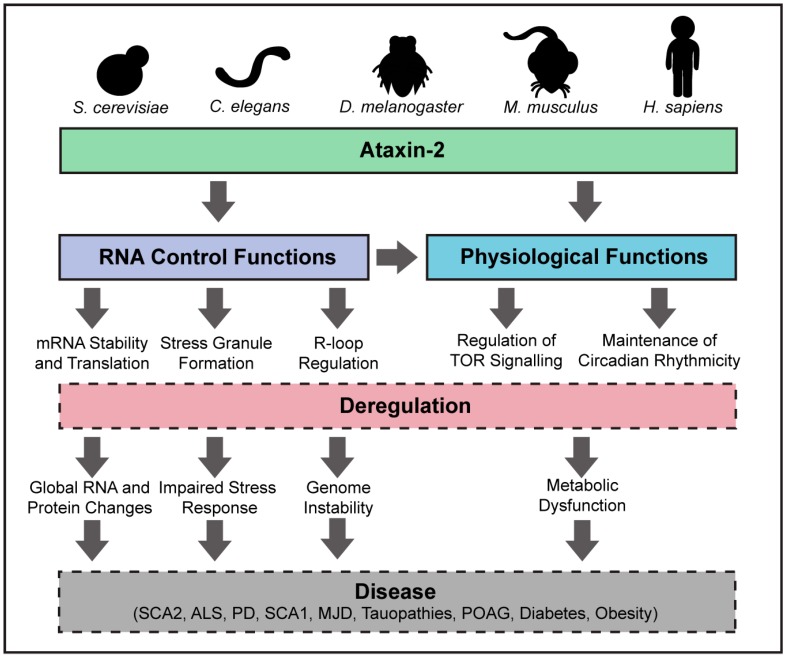
Multifaceted functions of Ataxin-2 and links to disease. Ataxin-2 functions to regulate several stages of RNA processing, with roles in physiological pathways. These functions include promoting mRNA stability and translation, as well as the regulation of R-loop and stress granule formation. These functions contribute to the control of metabolic pathways such as TOR and circadian rhythmicity. Deregulation of any of these processes can give rise to a wide range of cellular dysfunction, which can promote disease. SCA2, spinocerebellar ataxia type II; ALS, amyotrophic lateral sclerosis; PD, Parkinson’s disease; SCA1, spinocerebellar ataxia type I; MJD, Machado-Joseph’s disease; POAG, primary open angle glaucoma.

Taken together, these disease-focused studies reveal putative roles for ATXN2 in processes that are deregulated in several diseases including PD, SCA1, MJD, tauopathies, POAG and obesity/type I diabetes (Figure 3). As more studies are conducted into the mechanism(s) through which ATXN2 mutations may promote disease, we expect that our appreciation of the broad molecular and physiological impact of Ataxin-2 proteins in different species will continue to grow.

## 4. Concluding Remarks

Our summary of the Ataxin-2 literature points to key and evolutionarily conserved roles of these proteins under standard and stress conditions. Connections between these roles of Ataxin-2 proteins and various human diseases are slowly emerging. The involvement of ATXN2 in various human diseases is probably a reflection of the vast protein-protein, protein-RNA, and protein-DNA interaction networks in which Ataxin-2 proteins participate. Advanced understanding of the role of Ataxin-2 proteins in these molecular networks should reveal hidden secrets of the cell while also possibly pointing to novel therapeutic strategies that may stop or delay disease progression.

## 5. Conclusions

In conclusion, a large body of evidence shows that Ataxin-2 proteins have conserved roles in regulating mRNA stability and translation. Moreover, Ataxin-2 represses R-loop accumulation-driven genome instability in yeast and human cells. Overall, Ataxin-2 functions impact many metabolic processes and intersect with different disease-linked factors in human neurodegenerative diseases.

## Figures and Tables

**Table 1:** Functions of Ataxin-2 in various physiological processes.

Ataxin-2 Target	Species	Physiological Function of Ataxin-2	Implications of Ataxin-2 Dysregulation	Refs
TORC1 *	*Saccharomyces cerevisiae*, *Caenorhabditis elegans*, *Mus musculus*, *Homo sapiens*	Regulates TORC1-mediated nutrient responses via sequestration to stress granules	Possible defects in various TORC1-mediated metabolic pathways	[53,54,55,56]
PER1 *	*Drosophila melanogaster*	Regulates circadian rhythms via activating translation of PER1	Impaired circadian clock, specifically a longer period of constant darkness	[57,58]
Unknown	*C. elegans*	Early embryonic development	Embryos show abnormal morphology/do not undergo cell division	[59]
Caspase activation	*H. sapiens*	Targets neuroblastoma cells for apoptosis	Reduction in apoptotic activity	[60]
Endophilin-A1 & A2	*S. cerevisiae*, *H. sapiens*, *M. musculus*	Potential role(s) in plastin-associated pathways and receptor endocytosis	Possible implications in actin development and structure/alteration of the epidermal growth factor receptor (EGFR) internalization at the plasma membrane	[61,62]
Insulin Receptor (Insr)	*M. musculus*	Potential role in translation of Insr mRNA translation and/or regulation of Insr proteins	Obesity and infertility	[63]
pAktERES	*Drosophila*	Development of peripheral tissue by regulating the formation of endoplasmic reticulum exit sites (ERES) formation in larval fat body	Defects in ERES development and cellular growth	[64]
Grb2	*H. sapiens*	Role in cell proliferation by regulating Grb2 levels	Gain of function: increase in proliferation Loss of function: Impaired developmental processes	[65]
Ccr4 and Khd1	*S. cerevisiae*	Directly interacts with Rpl12a and Rpl12b proteins to regulate Ccr4 and Khd1 mediated cell growth	Possible growth defects	[66]
CZY-20	*C. elegans*	Regulates centrosome assembly and levels of centrosome-associated microtubules	Defects in cytokinesis—in severe cases Ataxin-2 implication can result in failure to undergo cytokinesis	[67]
PAR-5 and ZEN-4	*C. elegans*	Promotes cell division via regulation of PAR-5 which in turn modulates a cytokinesis pathway that targets ZEN-4 to the spindle midzone	Defects in spindle alignment and midzone assembly	[68]

* Physiological functions of Ataxin-2 discussed in detail in this review.
